# Longitudinal distribution of macroinvertebrates in snowmelt streams in northeast Greenland: understanding biophysical controls

**DOI:** 10.1007/s00300-017-2212-2

**Published:** 2017-10-20

**Authors:** C. L. Docherty, D. M. Hannah, T. Riis, S. Rosenhøj Leth, A. M. Milner

**Affiliations:** 1 School of Geography, Earth & Environmental Science, University of Birmingham, Edgbaston, Birmingham, B15 2TT UK; 2 Department of Atmospheric Environment and Aquatic Ecosystem, Institute of Mountain Science, Shinshu University, Matsumoto, 390-8621 Japan; 3 Department of Bioscience, Aarhus University, Aarhus, Denmark; 4 Institute of Arctic Biology, University of Alaska, Fairbanks, AK 99775 USA

**Keywords:** Chironomidae, Macroinvertebrates, Rivers, Zackenberg, Arctic

## Abstract

In a changing climate, Arctic streams are expected to show more influence from snowmelt, rainfall and groundwater, and less domination from glacial meltwater sources. Snowmelt streams are characteristic features of Arctic ecosystems, yet our current understanding of longitudinal patterns in benthic macroinvertebrate assemblages in these systems is limited when compared to glacier-fed systems. This study characterised longitudinal patterns of macroinvertebrate communities in snowmelt streams in northeast Greenland to provide novel insights into Arctic stream communities as dominant water sources shift with climate change. Benthic macroinvertebrates and environmental variables were sampled at three sites along five streams. Taxa diversity, evenness and abundance were expected to increase with distance from the stream source due to enhanced channel stability and warmer water temperature. This expectation for diversity and evenness was found in two streams, but abundance was up to ten times higher at the upstream sites compared to downstream, where biofilm biomass and ionic load were also highest. Here communities were largely dominated by the genus *Eukiefferiella* (Chironomidae). In the other three streams, no clear pattern in longitudinal macroinvertebrate community composition was evident due to low channel stability along the entire stream length. This study highlights the considerable variation in macroinvertebrate zonal distribution between snowmelt streams in northeast Greenland. A change towards more snowmelt-dominated streams in the Arctic could lead to shifts in the longitudinal organisation of macroinvertebrate community assemblages and the dominant species as a function of channel stability characteristics.

**Electronic supplementary material:**

The online version of this article (doi:10.1007/s00300-017-2212-2) contains supplementary material, which is available to authorized users.

## Introduction

Over the past 100 years, climate change has had a major impact on Arctic regions, with air temperature rise three times higher than the global average (2.9 °C compared to 0.8 °C, respectively) (Comiso and Hall 2014; Overland et al. 2015). Winter air temperature is predicted to further increase by 18 °C by the end of the century in northeast Greenland (Stendel et al. 2008) and with predicted increases in snowfall and rainfall, and decreased permafrost extent (Dyergerov and Meier 2000; White et al. 2007; Foster et al. 2008; Stendel et al. 2008) these changes are expected to significantly influence Arctic freshwater ecosystems. Both hydrology and thermal regimes (van Vliet et al. 2013) will be changed with effects on stream communities (Blaen et al. 2014; Chin et al. 2016). High Arctic streams are typically extreme habitats (Wharton 2002) characterised by low water temperature and high turbidity supporting specialised taxa able to tolerate these conditions, as observed in alpine regions (Snook and Milner 2001). Glaciers are principal water sources for Arctic streams as they are for alpine streams (Brown et al. 2003) but because of the retreat of glaciers across the region due to climate change, Arctic streams are expected to become less characterised by glacial runoff and snow will become the dominant meltwater source, along with increased rainfall and groundwater inputs, as predicted for alpine streams (Hannah et al. 2007). This shift in water source will potentially alter stream physicochemical habitat. Given that macroinvertebrate species have evolved specific adaptations and survival strategies (Danks 1971; Danks and Oliver 1972, Danks 1990; Danks et al. 1994; Danks 2004) to tolerate the harsh environmental conditions found in Arctic streams (including low water temperature, low channel stability, and limited food availability), changes in dominant stream water source are predicted to change macroinvertebrate community assemblages (Prowse et al. 2006; Anisimov et al. 2007; Milner et al. 2009; Blaen et al. 2014).

The Milner and Petts conceptual glacier-fed streams model (Milner and Petts 1994; Milner et al. 2001) was devised to predict longitudinal changes in macroinvertebrate communities downstream of a glacial source as a function of a changing physicochemical environment. The model states that glacier-fed streams have particularly deterministic physicochemical variables and macroinvertebrate assemblages in their headwaters that change with increasing distance from the glacier snout. This change allows for predictions to be made as to macroinvertebrate community composition given that some macroinvertebrate species are restricted to specific niches determined by certain environmental variables (Milner and Petts 1994; Lencioni and Rossaro 2005; Niedrist and Füreder 2016). The glacier-fed model characterised the primary environmental variables shaping macroinvertebrate communities along the length of a glacial stream to be downstream changes in; (1) maximum water temperature; and (2) channel disturbance regime where water becomes increasingly warm and stream channel becomes increasingly stable with distance from the source (Milner and Petts 1994; Milner et al. 2001). As macroinvertebrates develop adaptations to specific environmental conditions, stream reaches that undergo high levels of disturbance are expected to have low species richness characterised by a few high disturbance-tolerant species (Townsend et al. 1997; Lods-Crozet et al. 2001). Milner and Petts (1994) state that the Chironomidae subfamily Diamesinae will often be the only taxon located near the glacial snout in the harshest of environmental conditions, and will be found further downstream jointly with Orthocladiinae before being replaced by other taxa, when species diversity increases. Since its publication, the model has been tested extensively in different glacial environments. Whilst deviations have been noted due to unique situations and local biogeography, there have been many similarities found globally in glacial streams that match the predictions of the Milner and Petts model (Füreder et al. 2001; Gíslason et al. 2001; Maiolini and Lencioni 2001; Milner et al. 2001; Lods-Crozet et al. 2001; Hieber et al. 2005; Finn et al. 2010; Jacobsen et al. 2010; Kuhn et al. 2011; Hamerlik and Jacobsen 2012; Jacobsen et al. 2014).

Compared with glacier-fed streams, our understanding of longitudinal macroinvertebrate community changes in snowmelt streams is not extensive and it is uncertain whether models such as the Milner and Petts (1994) can be applied to these streams dominated by this water source. Snowmelt streams are characterised by having a wider water temperature range and marked diurnal variation, with maximum temperatures reaching 10 °C. The typical definition of snowmelt streams is that they generally have low turbidity, apart from during peak snowmelt, and have higher channel stability than glacial melt streams (Milner and Petts 1994; Ward 1994; Brown et al. 2003). This variability in habitat conditions can make snowmelt streams a challenging environment for macroinvertebrates. Longitudinal patterns in macroinvertebrate communities in glacial streams are deterministic due to the downstream decrease in glacial influence. Snowmelt streams are not so predictable (Ward 1994) because the presence of snowmelt inputs along the length of the stream acts as localised modifiers of physicochemical habitat. With the likely increase in snowmelt-fed streams in a warmer Arctic, it is vital we build a greater understanding of longitudinal changes on these systems.

To fill this research gap, we investigated longitudinal patterns in macroinvertebrate community composition in five snowmelt streams in northeast Greenland. Limited research has been conducted on Greenlandic stream ecology (but see Friberg et al. 2001; González-Bergonzoni et al. 2014 for studies from the west coast), and it is known to have a limited diversity of aquatic insects due to its geographical isolation and short time since deglaciation for establishment (Böcher et al. 2015). Our aim was to understand the longitudinal distribution of macroinvertebrates in snowmelt streams in northeast Greenland and their relation to environmental controls. The objectives of the study were to (1) explore changes in macroinvertebrate taxa richness, diversity and abundance with increasing distance from snowmelt sources in relation to environmental variables; (2) identify indicator taxa for specific habitats conditions; and (3) to compare changes with the glacier-fed rivers model to see if there are predictable patterns.

Given the harsh environmental conditions of snowmelt streams, we hypothesise that macroinvertebrate community assemblages in snowmelt streams will follow the general trend of the model for glacial streams (Milner et al. 2001), with higher taxa diversity, abundance, richness and evenness at sites furthest from the snowmelt source, where environmental conditions are more favourable.

## Methods

### Study area

**Fig. 1:**
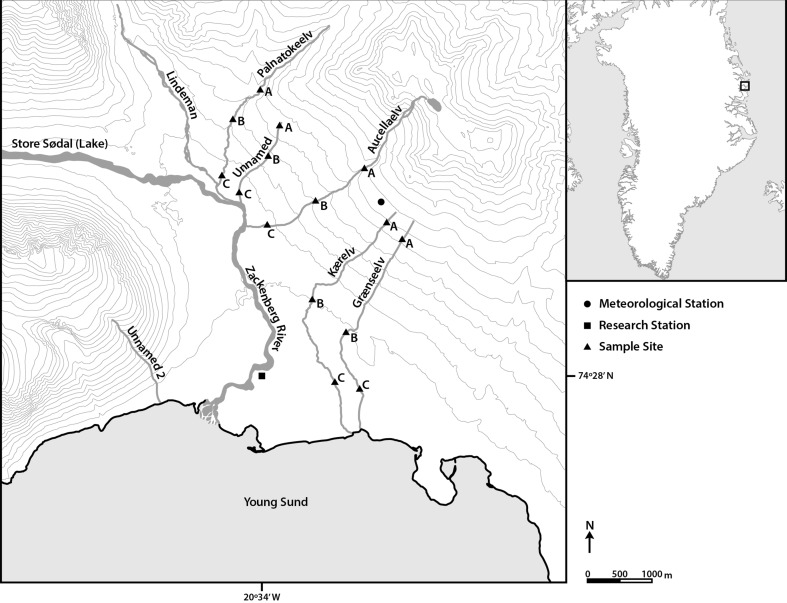
Map of the sampled stream systems around the Zackenberg research station with sites A, B and C. *Note* The large snowpack above Aucellaelv covered a large proportion of the upper reaches of the stream during the field campaign, as such, in the map the top sampling site appears far from the stream source; however, it was located directly below the main snowpack

The five streams studied were headwater streams in close proximity to the Zackenberg research station (74º28′N, 20º34′W) located within the northeast Greenland National Park in the high Arctic climatic zone (Fig. 1). The drainage basin is not connected to the Greenland ice sheet, which is at approximately 60-km distance. Altitude in the region varies between sea level and 1450 m a.s.l. and the low lands are characterised by wide valleys created through glacial erosion (Mernild et al. 2007). The region is characterised by continuous permafrost with active layer depth varying between 0.3 and 0.65 m (Christiansen et al. 2008). The underlying geology of the area is sedimentary.

Mean annual air temperature is −9.1 °C with the warmest month being July with a mean air temperature of 5.8 °C. Annual mean precipitation is 261 mm falling mainly as snow (Hansen et al. 2008) which is the principal water source for all five streams; however, Palnatokeelv and Aucellaelv also receive small glacier meltwater contributions. All streams originate in the mountains Aucellabjerg and Palnatokebjerg from snowpacks of varying sizes. During fieldwork, smaller snowmelt inputs were present along the length of the streams. We sampled three sites (Fig. 1, A–C) along each of the five study streams, and all fieldwork was undertaken between the 9th and the 14th July 2015. Due to the short field campaign, weather conditions could not be controlled and so some variation was experienced when sampling different sites. Weather conditions were mild and sunny when sampling Kaerelv and Aucellaelv, and cool and cloudy when sampling Graenseelv, Unnamed and Palnatokeelv. In all streams excluding Palnatokeelv, site A was immediately in front of the main snowpack, at the stream source. However, due to logistical difficulties, it was not possible to reach the source of Palnatokeelv and so site A in this stream was located at a lower longitudinal reach.

### Environmental variables

To characterise hydrological regimes and to verify change in environmental conditions along the stream continuum, physicochemical variables were measured at the same time as macroinvertebrate sampling occurred. Water temperature, conductivity and pH were measured using a waterproof HI-98129 Pocket EC/TDS and pH Tester (Hanna). Dissolved oxygen was measured using a YSI ProODO Optical Dissolved Oxygen meter. Discharge was calculated using the velocity-area method with a flow meter (μP-TAD Hӧntzsch instruments Germany). Water samples were collected to analyse for major ions and nutrients. NH_4_^+^, ${\text{NO}}_{3}^{ - }$ and ${\text{PO}}_{4}^{3 - }$ were analysed using the hypochlorite, cadmium reduction and ascorbic acid methods, respectively, on a Lachat QuikChem flow injection analyzer (Lachat Instruments, APC Bioscientific Limited, England; APHA 2012), and major ions were analysed on a ICP-MS (PerkinElmer Instruments, Optima 2000 DV). The Pfankuch Index was calculated for each site to determine channel stability (Pfankuch 1975) using all three components of the index (upper banks, lower banks and stream bed), where higher Pfankuch Index values correspond to lower channel stability. Suspended sediment samples were collected manually only at downstream sites (site C). Samples were filtered onto pre-weighed GF/F filter papers, dried at 60 °C for 48 h and then re-weighed. In order to measure stream bed sediment size (*D*_50_), 100 randomly selected stones or pebbles were measured along their widest section (B axis).

Chlorophyll *a* (Chl *a*) was measured as a proxy for benthic algae biomass. Five stone samples (> 6 cm) were collected from each site. Biofilm was removed from the stones using a toothbrush and was collected on a Whatman GF/C filter paper and frozen until analysis. The filter papers were then submerged in 96% ethanol and absorbance was measured at 665 and 750 nm on a spectrophotometer (UV 1700 Spectrophotometer, Shimadzu, Japan). Chl *a* biomass was calculated as

$$ \frac{{Chl a = \left( {Abs_{665} - Abs_{750} } \right) \times E}}{{83.4 \times A \times 10^{ - 4} }}, $$

where *E* is volume of ethanol (ml); 83.4 is the absorption of Chl *a* in ethanol; *A* is the sample area (cm^2^); and 10^−4^ is the conversion factor from cm^2^ to m^2^.

### Benthic macroinvertebrate sampling

Five replicate macroinvertebrate samples were collected randomly within a reach of approximately 20 m using a Surber sampler (0.1 m^2^ 300 µm mesh size) at each site. Samples were preserved in 90% ethanol in the field and stored in Whirlpak bags until identification. In the laboratory, samples were rinsed through a 200-μm sieve and sorted under ×10 magnification. Subsampling was carried out on samples from Kaerelv site A and Graenseelv site A, which supported the highest densities of Chironomidae. Chironomids with dark head capsules were immersed in 10% potassium hydroxide (KOH) solution on a hot plate at 60 °C, for 15 min to lighten the head capsule and to make characteristic features easier to see. They were then mounted on slides using dimethyl hydantoin formaldehyde (Steedman 1958) (DMFH) mountant. Chironomidae were identified to species-group or type using the following keys; Cranston (1982), Wiederholm (1983), Brooks et al. (2007), Ferrington and Sæther (2011) and Lindegaard (2015). Oligochaeta were identified only to subclass, Collembola to order, and other taxa were identified to family or subfamily level using the keys Nilsson (1997) and Dobson (2013). Table S1 in supporting material provides a taxa list.

### Data analysis

In order to visualise similarities and dissimilarities between sites, a non-metric multidimensional scaling (NMDS) analysis was compiled with the Bray–Curtis dissimilarity coefficient. Macroinvertebrate data were square root transformed due to the small proportion of large values distorting distribution. Log_10_ transformed environmental variables were fitted to the ordination after 999 permutations and significant variables were plotted in the ordination space. To describe downstream changes in community composition, Bray–Curtis dissimilarity was calculated on log transformed data. One-way ANOVA was used to determine significant differences in community measurements between the three sites for each stream. Spearman’s Rank correlation coefficients were used to determine the correlation between different community measurements and the environmental variables determined significant through the NMDS. Spearman’s Rank was used due to the small size of the data set and potential for non-linear relationships (Zar 2010). Cluster analysis was calculated for community similarity between sites in the R environment using the complete method distances of Bray–Curtis similarity index and square root transformed data. A two-way cluster analysis between sites and taxa similarity was constructed in the PAST software using paired-group method with Bray–Curtis similarity index. Sites were grouped by taxa abundance and taxa were grouped by abundance in different streams. Rare taxa (< 5%) were excluded to avoid large influence on analysis by low abundance taxa (e.g. Niedrist and Füreder 2016). Indicator taxa were determined for specific habitats using the labdsv function in *R*.

## Results

### Environmental conditions

Environmental variables varied spatially along the longitudinal gradient and between streams (Table 1). Graenseelv sites B and C were at a lower altitude than sites B and C in other streams, whilst site C of Unnamed was at a higher altitude than site C in other streams. Discharge and water temperature increased downstream except in Aucellaelv. No marked longitudinal patterns in conductivity were evident but was notably higher in Aucellaelv (mean 89.3 ± 3.40 μS *n* = 3) compared to all other streams (mean varied between 23.67 ± 1.7 μS and 27.33 ± 4.11 μS *n* = 3). Channel stability was highest at downstream sites in Kaerelv and Graenseelv with the highest channel stability recorded at Kaerelv B (Pfankuch Index: 70). No defined pattern in channel stability with distance from the source was found in the other three streams. Channel stability was lower in Unnamed, Palnatokeelv and Aucellaelv where all sites scored between 111 and 124 (excluding Unnamed site B which scored 85), and displayed a high degree of channel mobility. Although suspended sediment concentration was only measured at the lower reaches of each stream, marked differences existed between streams, with Kaerelv, Graenseelv and Unnamed having low concentrations (0.5–7.3 mg L^−1^) compared to Palnatokeelv (96 mg L^−1^) and Aucellaelv (1120 mg L^−1^).

**Table 1:** Stream physicochemical properties in three sites in five study streams in Zackenberg area in Northeast Greenland

Stream	Site	Elevation	Approximate distance from site A	Pfankuch Index	pH	Water temperature	Conductivity	DO	Discharge	Suspended sediment	Sediment D_50_	Chl *a*
		m.a.s.l	m			°C	µs cm^−1^	%	L s^−1^	mg L^−1^	mm	μg m^2^
Kaerelv	A	179	0	93	7.0	2.7	26	82.4	190	–	113.8	8241.8
	B	102	1560	70	6.9	6.3	36	92.0	235	–	71.1	3172.7
	C	47	3040	74	7.0	9.4	36	76.5	316	5.1	51.2	392.7
Graenseelv	A	125	0	104	7.4	2.3	32	79.1	177	–	196.5	3096.4
	B	46	1340	83	7.2	3.4	34	81.2	186	–	115.4	1301.6
	C	19	2570	78	7.1	4.4	32	78.0	199	7.3	33.9	198.5
Unnamed	A	193	0	116	7.6	2.8	38	79.4	69	–	87.5	267.8
	B	136	500	85	7.2	2.2	32	79.9	68	–	76.0	266.4
	C	113	1234	113	7.0	2.3	42	88.0	376	0.5	90.3	504.0
Palnatokeelv	A	137	0	116	7.6	3.1	23	78.7	–	–	246.1	234.2
	B	124	707	124	7.3	4.2	22	74.5	–	–	151.6	562.9
	C	56	1429	114	7.2	5.8	26	77.8	788	96.3	100.7	544.6
Aucellaelv	A	185	0	119	7.4	2.6	86	74.1	631	–	193.2	134.4
	B	101	881	111	7.2	4.0	94	73.8	976	–	156.5	341.2
	C	68	1678	116	7.0	5.3	88	75.1	646	1120.3	96.5	289.9

Stream bed substrate decreased in size with distance from the source in all streams except Unnamed, where lowest size was situated at the middle sampling site. Biofilm biomass was highly variable. Unnamed and Palnatokeelv showed increasing biomass with distance from the source, where as Kaerelv and Graenseelv showed exceptionally high biofilm biomass at upstream sites that gradually decreased with increasing distance downstream (Table 1).

Si concentration was highest in Kaerelv and Graenseelv compared to Unnamed, Palnatokeelv and Aucellaelv and tended to be highest at upstream sites, whilst ${\text{NO}}_{3}^{ - }$ was lowest in Kaerelv, Graenseelv and Unnamed. ${\text{PO}}_{4}^{3 - }$ concentration decreased with increasing distance from the water source but NH_4_^+^ concentration showed no pattern (Table 2).

**Table 2:** Water chemistry data in three sites in five study streams in Zackenberg area in northeast Greenland

Stream	Site	Si	${\text{NH}}_{4}^{ + }$	${\text{NO}}_{3}^{ - }$	${\text{PO}}_{4}^{3 - }$
		Mg L^−1^	μg L^−1^	μg L^−1^	μg L^−1^
Kaerelv	A	1.47	11	19	7
	B	1.17	30	19	7
	C	1.28	15	6	3
Graenseelv	A	1.57	18	18	7
	B	1.35	11	9	3
	C	1.42	55	3	3
Unnamed	A	1.01	43	8	4
	B	0.72	11	5	2
	C	0.95	13	9	3
Palnatokeelv	A	1.14	48	49	9
	B	1.19	21	22	5
	C	0.95	19	43	3
Aucellaev	A	1.02	20	31	11
	B	0.94	15	37	4
	C	0.80	19	27	5

### Macroinvertebrate community assemblages

In total, 3103 individuals were identified (37 taxa) of which 94% were Chironomidae, encompassing 29 taxa. Eight taxa were found only at sites B and C, whilst only one taxon was found exclusively at the uppermost site A (*Chaetocladius dentiforceps*-*type*).

**Fig. 2:**
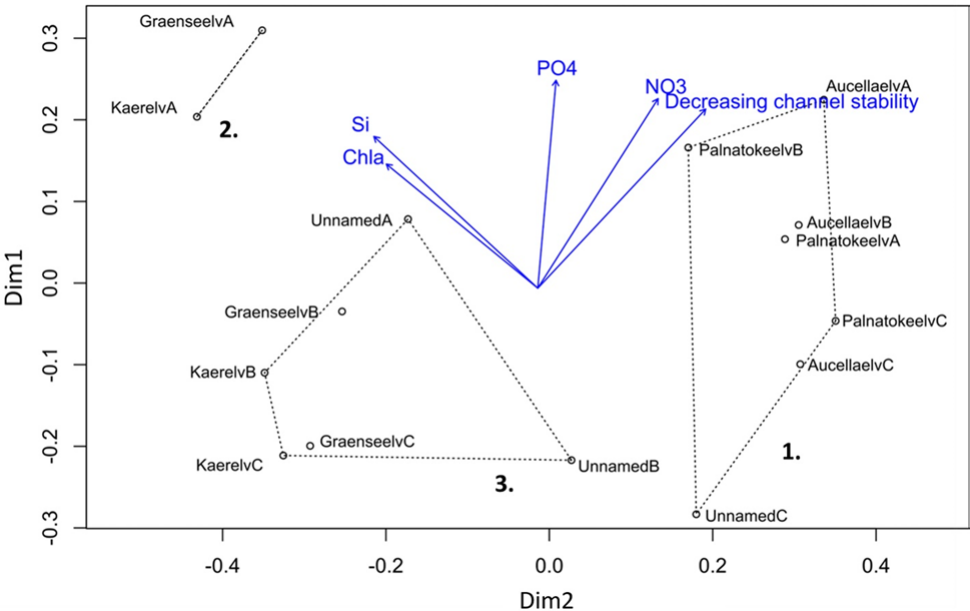
NMDS of sites using abundance of macroinvertebrate taxa. Dimensions 2, stress 0.113. Groups 1–3 are marked and correspond to groups 1–3 in Fig. 3. Sites were always sampled from upstream (site A) to downstream (site C)

**Fig. 3:**
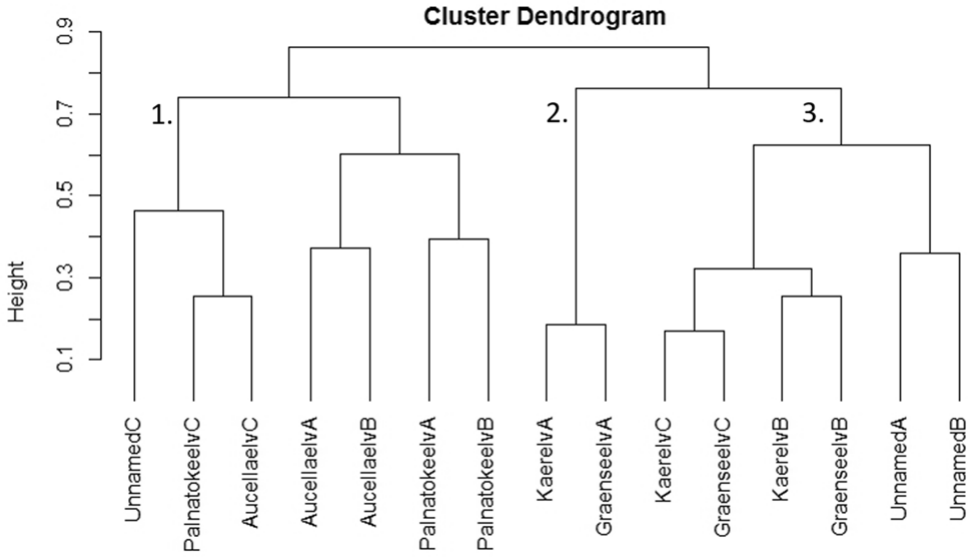
Dendrogram based on abundance of macroinvertebrate taxa. Cophenetic correlation 0.814. Groups 1–3 are marked and correspond to group 1–3 in Fig. 2

**Fig. 4:**
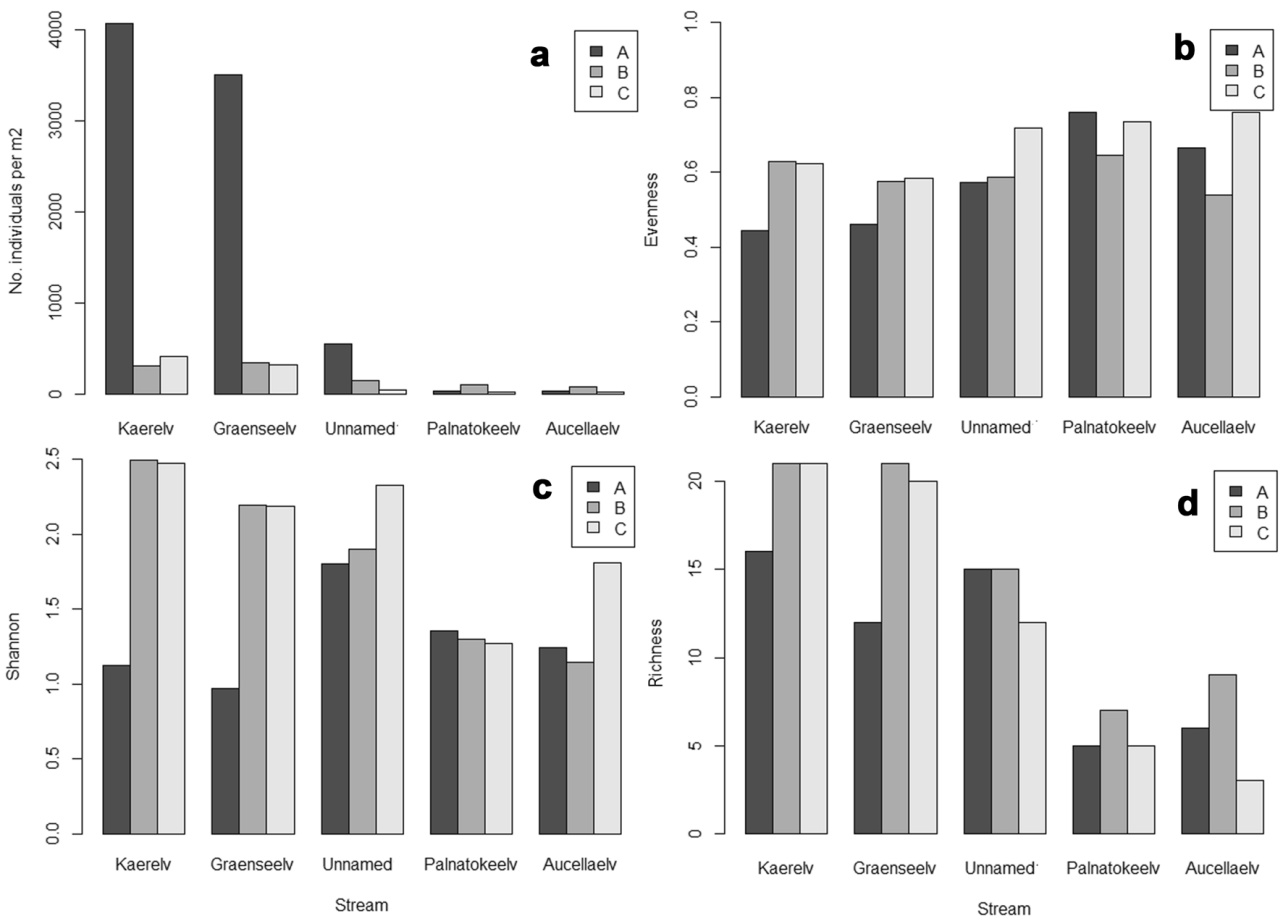
Macroinvertebrate community composition measurements: **a** Macroinvertebrate abundance, **b** evenness, **c** Shannon’s diversity, **d** richness

**Fig. 5:**
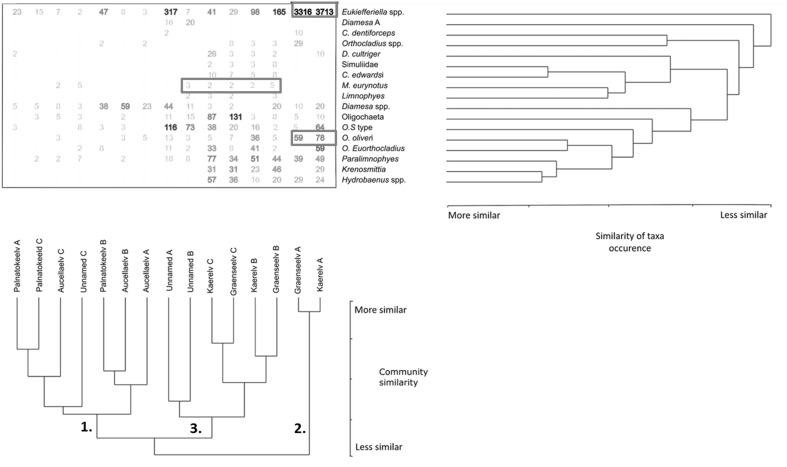
Two-way cluster analysis of most abundant taxa (top right) and the 15 stream sites (bottom). Data matrix gives taxa abundance (individuals m^2^) at each site

Both the NMDS and the cluster analysis divided the study sites into three groups (Figs. 2, 3). The first group consisted of all Palnatokeelv and Aucellaelv sites along with Unnamed site C. This group was characterised by low channel stability and high nutrient concentration (Table 3), although this was variable within the group with upstream sites having lowest channel stability and highest nutrient concentration. Macroinvertebrate abundance was low in these streams, and Aucellaelv and Palnatokeelv displayed no clear patterns within streams in abundance, evenness, diversity or taxa richness (Fig. 4). No indicator taxa were identified for this group although the genera *Diamesa* and *Eukiefferiella* were the most common taxa (Fig. 5). Group 2 consisted of Kaerelv and Graenseelv upstream sites and were characterised by high Chl *a* and high Si concentrations (Figs. 2, 3). Macroinvertebrate abundance was exceptionally high at these sites but evenness and diversity were low (Fig. 4). Indicator taxa were the genus *Eukiefferiella* (indicator value 82%, *p* = 0.009) and *Orthocladius oliveri*-type (indicator value 68%, *p* = 0.014) (Fig. 5; Table 4). *Eukiefferiella* were found in very large numbers at these two sites (3713 m^2^ in Kaerelv and 3316 m^2^ in Graenseelv) and were found to be highly correlated with Chl *a* density (Spearman rank correlation: *rs* = 0.61, *n* = 15, *p* = 0.02). The third group consisted of sites B and C in Kaerelv and Graneseelv and sites A and B in Unnamed (Figs. 2, 3). This group was characterised by high channel stability and low Chl *a* concentration. Sites in Kaerelv and Graenseelv in this group had the highest macroinvertebrate evenness, diversity and taxa richness of all the streams (Fig. 4). One indicator taxon was identified for this group and that was the species group *Metriocnemus eurynotus* (indicator value 60%, *p* = 0.047).

**Table 3:** Significant correlations for environmental variables in the ordination space

Variable	*R* ^2^	*p*
Channel stability	0.77	0.001
Chl *a*	0.49	0.019
Si	0.64	0.002
NO_3_^−^	0.65	0.001
PO_4_^3−^	0.56	0.018

**Table 4:** Indicator taxa for different habitats

Cluster	Principal streams	Habitat type	Species group	Indicator value (%)	*p* value
2	Kaerelv Graenseelv	Upstream sites. Close proximity to snowpack source. Low channel stability. Low suspended sediment.	*Eukiefferiella*	82	0.009
2			*Orthocladius oliveri*	68	0.014
3	Kaerelv Graenseelv	Downstream sites. Stable channels and warmer water temperatures.	*Metriocnemus eurynotus*	60	0.047

Longitudinal patterns were visible in Kaerelv, Graenseelv and to a lesser extent Unnamed as can be seen by the NMDS and dendrograms (Figs. 2, 3, 5). In Unnamed, abundance was much lower at site A compared to the respective sites in Kaerelv and Graenselv, but decreased gradually between the three sites (Fig. 4a). In Kaerelv, Graenseelv and Unnamed, evenness, diversity and richness were lowest at site A compared to sites B and C (Fig. 4b–d).

**Fig. 6:**
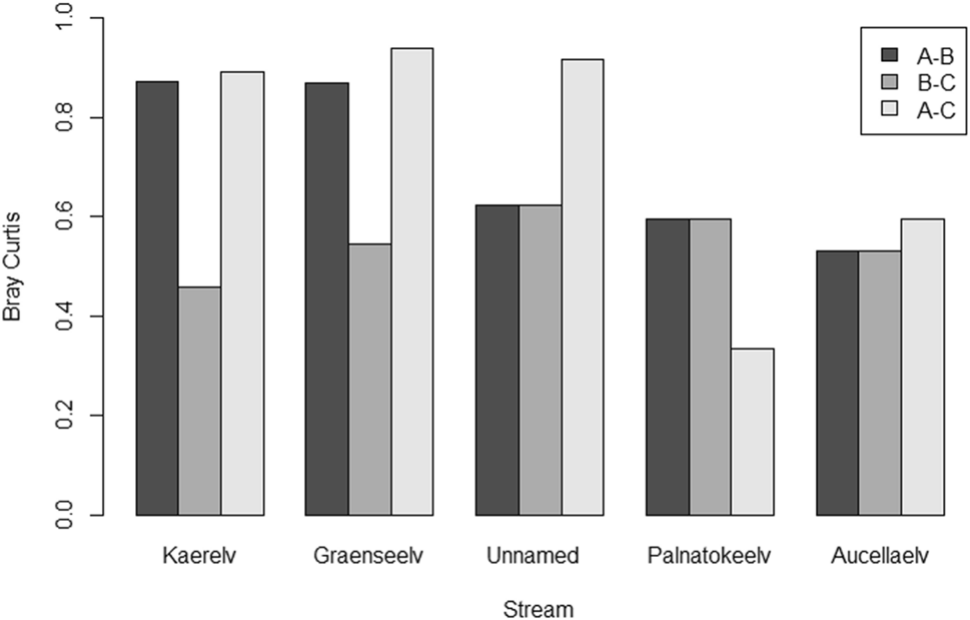
Bray Curtis dissimilarity between sites A and B, sites B and C and sites A and C

In Kaerelv and Graenseelv, site A was significantly different from sites B and C for density, evenness and diversity. Evenness was significantly different in sites A and B of Unnamed compared to site C (Table 5). Kaerelv and Graenseelv showed large differences in terms of the community composition between sites A and B, whilst sites B and C were more similar. This is in contrast to Unnamed, Palnatokeelv and Aucellaelv where sites B–C show the largest variation (Fig. 6).

**Table 5:** One-way ANOVA results comparing longitudinal sites

Stream	Density	Evenness	Shannon	Richness
Kaerelv	*F*(1,2) = 1575.9, *p* = 0.016	*F*(1,2) = 1697.2, *p* = 0.015	*F*(1,2) = 6144.519, *p* = 0.008	–
Graenseelv	*F*(1,2) = 41,621.95, *p* = 0.003	*F*(1,2) = 317.12, *p* = 0.036	*F*(1,2) = 45,226.5, *p* = 0.003	–
Unnamed	–	F(1,2) = 158.4, *p* = 0.050*	–	–
Palnatokeelv	–	–	–	–
Aucellaelv	–	–	_–_	–

All significant sites, site A is different to sites B and C, however * significant difference between sites A and B with site C

Channel stability and Si concentration were found to be the most significant environmental variables for Kaerelv and Graenseelv macroinvertebrate community composition, where there is a negative relationship with evenness, Shannon diversity and taxa richness (Table 6). Due to the method used to measure channel stability, these results signify an increase in stability leading to an increase in evenness, diversity and richness. However, when all streams were tested together, Si concentration was shown to be significantly correlated with density (Spearman rank correlation *rs* = 0.80, *n* = 15, *p* = 0.0004) and taxa richness (Spearman rank correlation *rs* = 0.70, *n* = 15, *p* = 0.0037) and for channel stability to be significantly correlated with taxa richness (Spearman rank correlation *rs* = −0.66, *n* = *15, p* = 0.008) (Table 6).

**Table 6:** Spearman’s rank correlation coefficients for community measurements with significant environmental variables

	Kaerelv and Graenseelv								All streams							
	Density		Evenness		Shannon		Richness		Density		Evenness		Shannon		Richness	
	*rs*	*p*	*rs*	*p*	*rs*	*p*	*rs*	*p*	*rs*	*p*	*rs*	*p*	*rs*	*p*	*rs*	*p*
Channel stability	0.77	**0.103**	−**0.93**	**0.008**	−**0.94**	**0.017**	−**0.81**	**0.046**	−0.48	0.073	0.43	0.107	−0.34	**0.220**	−**0.66**	**0.008**
Chl *a*	0.37	0.497	−0.32	0.538	−0.14	0.803	−0.27	0.600	0.39	0.149	−0.33	0.223	0.06	0.822	0.42	0.117
Si	0.71	**0.136**	−**0.93**	**0.008**	−**1**	**0.0028**	−**0.94**	**0.005**	**0.80**	0.0004	−0.48	0.068	0.02	**0.929**	**0.70**	**0.004**
${\text{NO}}_{3}^{ - }$	0.09	0.919	−0.03	0.957	0.09	0.919	−0.09	0.863	0.80	0.723	0.45	0.093	0.09	0.763	0.16	0.578
${\text{PO}}_{4}^{3 - }$	0.20	0.714	−0.41	0.425	−0.31	0.564	−0.52	0.294	0.18	0.511	0.09	0.737	0.34	0.221	0.05	0.859

*n* = 15 for all sites. *n* = 6 for Kaerelv and Graenseelv correlations

Bold text indicates significant correlations

## Discussion

This study provides new insights into the macroinvertebrate community composition dynamics in snowmelt streams in northeast Greenland, which is vital as snow becomes an increasingly important water source for streams in the Arctic as glacial influence becomes reduced.

### Landscape scale patterns in habitat and macroinvertebrate community assemblages

This study found a marked difference in macroinvertebrate community structure between streams, with Kaerelv and Graenseelv supporting high taxa richness and diversity whereas in Palnatokeelv and Aucellaelv these metrics were low, and intermediate in Unnamed. Unlike the typical definition for snowmelt streams, the streams in this study displayed low water temperature variation between sites, whereas channel stability was highly variable. The low temperature variation between sites was found even though weather conditions varied between the days when samples were collected. As such, the different weather conditions are thought to have minimal impact on the results of this study. Palnatokeelv and Aucellaelv were characterised by very high levels of suspended sediment (96.3 and 1120.3 mg L^−1^, respectively), and low channel stability with a high degree of channel mobility along the entire stream length (Docherty et al. 2017). Despite these two streams having small headwater glaciers located within their catchments, glacial inputs were minimal due to the large snowpacks present and the early field campaign before peak glacier melt. This means they fitted the classification of Brown et al. (2003) as nival streams. Here, we propose that the low channel stability of Palnatokeelv and Aucellaelv was caused by the larger snowpacks at the stream source leading to large spring floods during the initial melt season destabilising floodplain sediments. Increased nivation processes weathering soils underneath snowpacks and in the surrounding areas of summer snowpack retreat (Christiansen 1998), creating stream sediments during melting and altering the habitat from what is typically defined as a snowmelt stream. The high nutrient load in Palnatokeelv and Aucellaelv is likely to be from in-stream weathering of suspended sediment (Chin et al. 2016). These low stability systems typically support low macroinvertebrate abundance as found in previous research on highly disturbed stream environments (Chin et al. 2016) dominated by the chironomids *Diamesa*, a genus typical of harsh glacial streams, and *Eukiefferiella*.

### Longitudinal patterns in habitat and macroinvertebrate community assemblages

Palnatokeelv, Aucellaelv and Unnamed did not show any deterministic longitudinal patterns and proved to be largely unpredictable in terms of their patterns in longitudinal macroinvertebrate community assemblages. For Palnatokeelv, this could be due to the location of the upstream site being a relatively large distance downstream of the source snowpack, making environmental conditions at the upstream site different compared to the other sites studied. Kaerelv and Graenseelv followed the predicted longitudinal patterns of higher diversity and richness at sites downstream from the source, similar to glacier-fed rivers, as well as in environmental conditions, with site A being significantly different from the other two sites (Milner and Petts 1994; Milner et al. 2001; Jacobsen et al. 2014). However, the marked decrease in macroinvertebrate abundance downstream from the water source was not expected as previous research on glacial streams shows macroinvertebrate abundance to increase with distance from the source or for there to be no clear pattern (e.g. Milner and Petts 1994; Ward 1994; Gíslason et al. 2001; Blaen et al. 2014). The high abundance of macroinvertebrates was significantly correlated with high biofilm biomass at upstream sites of Kaerelv and Graenseelv. Previous work on these streams suggests that N is a main limiting nutrient in these systems and that biofilm biomass increases with N additions (Docherty et al. in press) and that diatom growth is determined by Si concentrations (Sabater and Roca 1990). In the majority of our study streams, especially Kaerelv and Graenseelv, Si and ${\text{NO}}_{3}^{ - }$ concentrations were highest at the upstream sites. This was due to discharge being lowest at upstream sites, creating more highly concentrated solutes, as well as the weathering action of snowpacks causing increased Si concentrations, and the preferential elution of ions from snowpacks (Johannessen and Henriksen 1978; Tranter et al. 1987; Helliwell et al. 1998; Brown et al. 2003). This causes larger ${\text{NO}}_{3}^{ - }$ inputs to the stream, compared to further downstream, through snowmelt runoff (Robinson et al. 2001; Hodson et al. 2002; Brown et al. 2003). This led to increased biofilm production and an ample food source for chironomids.

*Eukiefferiella brehmi*-group was the dominant species group at upstream sites in Kaerelv, Graenseelv and Unnamed. Whilst this is usually classified as a collector-gatherer, feeding on deposited sediment, it has also been described as a scraper, shearing food from the surface of rocks (Armitage et al. 1995; Tavares-Cromar and Dudley Williams 1997). In general, many species of chironomids show flexibility in food resource preference, and as such are able to adapt to food resources available within the local environment (Armitage et al. 1995 and references therein) and the genus *Eukieffereilla* has been described as an opportunistic taxon (Herbst and Cooper 2010). *Eukiefferiella* in these systems typically fills a niche that excludes many other taxa, which are restricted to downstream regions. However, upstream sites in Kaerelv and Graenseelv were characterised by low water temperature and channel stability, which are known to limit primary productivity and macroinvertebrate growth, and to affect the ability of algae and invertebrates to attach to substrate (Milner and Petts 1994; Brown et al. 2003). These habitat characteristics are typical of upstream sites in glacier melt streams that are typically dominated by the genus *Diamesa*. However, *Diamesa* may be restricted to low channel stability habitats as they are competitively excluded from more preferable habitat where other taxa are able to colonise (Flory and Milner 2000). The ionic enrichment at upstream sites characteristic of snowmelt appears to create suitable habitat conditions to support high densities of benthic algae to grow, creating a refugia and supporting large macroinvertebrate abundances, namely of the genus *Eukiefferiella*, which then excludes *Diamesa* from the community assemblage. Other studies have found *Eukiefferiella* in European glacier-fed streams but at further distance from the source, inhabiting unstable channels within 200 m of glacial snouts alongside *Diamesa* spp. (Brittain et al. 2001; Gíslason et al. 2001; Lods-Crozet et al. 2001). However, in New Zealand glacial streams where *Diamesa* are absent, *Eukiefferiella* are co-dominant with *Maoridiamesa* in close proximity to the glacier snout along with two *Deleatidium* an ephemeropteran species (Cadbury et al. 2011).

With increasing distance from the source in Kaerelv and Graenseelv, biofilm biomass and macroinvertebrate abundance decreased; however, the increase in channel stability led to an increase in macroinvertebrate richness, diversity and community evenness, typical of glacier-fed streams (e.g. Milner and Petts 1994; Ward 1994; Gíslason et al. 2001; Blaen et al. 2014). The results follow previous studies on glacial streams, showing water temperature to increase downstream (Milner et al. 2001; Jacobsen et al. 2010; Kuhn et al. 2011; Jacobsen et al. 2014); however as temperature was recorded only by spot tests, we could not determine the importance of this in influencing macroinvertebrate communities. The variation in temperature between sites could also be due to variation in the time of day when measurements were taken. Reductions in ${\text{NO}}_{3}^{ - }$ and Si associated with decreased meltwater inputs and higher discharge diluting ion concentrations resulted in fewer resources available for biofilm colonisation and so, notably reduced biofilm biomass compared to upstream sites. However, the increase in channel stability provided a suitable habitat for more diverse macroinvertebrate assemblages to form (Milner and Petts 1994).

### Insights to indicator taxa

The three indicator taxa identified in this study represent two different habitat types. *M. eurynotus*-type was characteristic of high stability stream reaches and formed part of a macroinvertebrate community characterised by high taxa richness, diversity and evenness. This finding was in contrast to research by Snook and Milner (2001) where *Metriocnemus* was most commonly found in high-stress habitats. It is possible that the individuals were different species of *Metriocnemus* in the two cases, or it could be a result of chironomid flexibility in feeding preferences, where they are able to adjust to different environmental conditions available. The other two indicator taxa were *Eukiefferiella* and *Orthocladius oliveri*-*type.* These were characteristic of upstream sites in the stable streams that had low channel stability but high nutrient and biofilm biomass. They showed high levels of stress tolerance in accordance with previous research on the taxa (Kownacki and Zosidze 1980; Gíslason et al. 2001; Lods-Crozet et al. 2001; Cadbury et al. 2011).

### Implications of global change

A reduction in glacial cover caused by a warming Arctic, combined with increased snowfall in some regions, will result in streams to become more influenced by snowmelt, as well as rainfall and groundwater, as is also being experienced in alpine regions (Hannah et al. 2007; Kattsov et al. 2007; Stendel et al. 2008; Dankers and Middelkoop 2008; Collins et al. 2013). Because of this, it is vital we have a full understanding of snowmelt stream dynamics, incorporating hydrological processes and ecological community composition, in order to make predictions for how Arctic streams will change in the future. Zackenberg provides an interesting base for research on snowmelt streams due to the low water temperature variability but high variation in channel stability—the two factors identified as being most influential to glacial stream community assemblages—providing a diversity of snowmelt streams that are both characteristic and uncharacteristic in their habitat. With reduced glacial extent and increased snowfall predicted in many areas of the Arctic, testing these findings in other Arctic regions is essential to understand patterns in snowmelt stream geomorphology and physicochemical habitat, and in macroinvertebrate community composition. This will allow us to better predict future changes.

A shift away from glacial streams towards more snowmelt-dominated streams could have two different outcomes for macroinvertebrate community composition along the stream length depending on local snowpack size and local geology. (1) In streams that meet the definition of typical snowmelt streams, higher nutrient inputs are to be expected through ionic enrichment with snowmelt, and increased benthic algae biomass at upstream sites. Macroinvertebrate community composition patterns could remain the same for taxa richness, evenness and diversity, but reverse for abundance with highest abundance at upstream sites, as well as see different species types present in macroinvertebrate communities. This includes the possible local extinction of typical glacial coloniser taxa such as *Diamesa*. Or; (2) low stability snowmelt streams characterised by their large snowpacks and unconsolidated soils could lead to a loss of macroinvertebrate zonation along the stream length, with low abundance and diversity typical, but with taxa composition more similar to glacial streams. Although these streams have high ionic loads due to high weathering rates, the low channel stability prevents nutrient uptake.

The findings from the streams in northeast Greenland, combined with previous research highlighting the unpredictability of snowmelt stream habitat and ecological zonation, draw attention to the considerable variation in macroinvertebrate distribution and community composition in snowmelt streams across northeast Greenland and the Arctic. However, despite these variations, patterns in community composition have been observed in these systems and indicator taxa identified for some of the streams. To improve the quality of these findings, research should be conducted over a longer timespan to represent the zonation patterns during the whole summer season and to capture inter-annual variation.

### Future research directions

The large differences between Kaerelv and Graenseelv with Unnamed, Palnatokeelv and Aucellaelv highlight the complexity and diversity in snowmelt stream habitat conditions with snowpack size and local geology influencing stream physicochemical habitat. This provides new aspects to consider when defining Arctic and alpine stream types and could be used to build on from the work of Brown et al. (2003) to divide the definition of nival stream systems into two new categories.

In relation to the Milner and Petts (1994) for glacial streams, this study has found clear differences compared to glacial systems as macroinvertebrate community assemblages are not deterministic in all snowmelt streams as was determined by Ward (1994). Streams with large snowpacks, showing low channel stability and high suspended sediment concentration throughout their lengths, leading to low macroinvertebrate abundance, diversity and taxa richness along their entire lengths. However, the streams in this study that best fit the definition of a typical snowmelt stream had certain characteristics which did fit the Milner & Petts model, such as increased diversity and taxa richness downstream along with increased channel stability.

The differences in length of streams and the distance between sites in the five streams could influence the results given the site furthest from the source in a short stream will be more influenced by source water conditions than the corresponding site in a longer stream. As such, this should be taken into account and incorporated into future studies.

Future directions of research should include increased taxonomic resolution, ideally through DNA barcoding, to be able to compare between sites on a species level. Replicating this study on snowmelt streams in other Arctic regions would be beneficial and would provide a broader understanding of macroinvertebrate communities in snowmelt streams, as would replicating this study in longer stream systems. To understand temporal changes and to infer how a changing climate could affect these systems, it is necessary to conduct long-term studies over the whole summer period linking stream habitat conditions and macroinvertebrate community composition to local weather conditions. Finally, to gain a full understanding of ecosystem scale processes on a longitudinal scale, it would be useful to study longitudinal nutrient spiralling dynamics and primary productivity within snowmelt streams, allowing us to determine the most biogeochemically active reaches of snowmelt streams.

## Electronic supplementary material

Below is the link to the electronic supplementary material.

Supplementary material 1 (DOCX 19 kb)
