# Supplementary material for: Longitudinal distribution of macroinvertebrates in snowmelt streams in northeast Greenland: understanding biophysical controls
Source: Polar Biol. 2017 Oct 20;41(8):1567–80. doi: 10.1007/s00300-017-2212-2 (PMC6428397; doi:10.1007/s00300-017-2212-2)
Supplement: Supplementary file 1 — Supplementary material 1 (DOCX 19 kb) [file 300_2017_2212_MOESM1_ESM.docx]

**Table S1. List of identified taxa**

| **Taxa** | **Identification literature** | **Number of specimens per site and stream*** |
| --- | --- | --- |
| **Chironomidae** |  |  |
| **Diamesinae** |  |  |
| *Diamesa* | Brooks 2007; Wiederholm 1983 | 4,0,2; 2,12,1; 27,7,2; 14,36,5; 3,23,3 |
| *Diamesa A.* |  | 0,0,0; 0,0,0; 10,12,0; 0,0,0; 0,0,0 |
| **Orthocladinae** |  |  |
| *Chaetocladius dentiforceps-*type | Brooks et al. 2007 | 0,0,0; 2,0,0; 1,0,0; 0,0,0; 0,0,1 |
| *Chaetocladius piger-* type | Brooks et al. 2007 | 2,0,1; 0,0,0; 0,0,0; 1,0,0; 0,1,0; |
| *Corynoneura edwardsi- type* | Lindegaard 2015; Wiederholm 1983 | 0,3,6; 0,5,4; 0,0,0; 0,0,0; 0,0,0 |
| *Cricotopus* type P | Brooks et al. 2007 | 0,0,0; 0,0,1; 0,0,0; 1,0,0; 0,0,0 |
| *Diplocladius cultriger* | Lindegaard 2015; Brooks et al. 2007 | 2,2,16; 0,0,2; 0,0,0; 0,0,0; 1,0,0 |
| *Eukiefferiella* | Wiederholm 1983 | 423,33,15; 324,38,11; 127,1,0; 0,4,3; 4,2,0 |
| *Eukiefferiella brehmi* group | Wiederholm 1983 | 335,27,10; 353,63,7; 67,3,0; 2,1,1; 10,27,9 |
| *Eukiefferiella gracei* group | Wiederholm 1983 | 0,0,0; 0,1,0; 0,0,0; 0,0,0; 0,0,0 |
| *Hydrobaenus conformis* type | Brooks et al. 2007; Sæther 1976 | 1,0,1; 0,0,1; 0,0,0; 0,0,0; 0,0,0 |
| *Hydrobaenus lugubris* type | Brooks et al. 2007; Sæther 1976 | 3,5,25; 5,10,15; 0,0,0; 0,0,0; 0,0,0 |
| *Krenosmittia* | Lindegaard 2015; Brooks et al. 2007; Wiederholm 1983 | 6,14,19; 0,28,19; 0,0,0; 0,0,0; 0,0,0 |
| *Limnophyes* | Wiederholm 1983 | 0,0,2; 0,2,1; 0,1,0; 0,0,0; 0,0,0 |
| *Metriocnemus eurynotus (=hygropetricus)-* type | Wiederholm 1983 | 0,3,1; 0,0,1; 0,2,3; 0,0,1; 0,0,0 |
| *Orthocladius Euorthocladius* | Cranston 1982 | 12,25,20; 0,1,5; 7,0,1; 0,0,0; 0,5,0 |
| *Orthocladius oliveri* type | Brooks et al. 2007 | 16,22,3; 12,3,4; 8,2,0; 3,2,1; 0,0,0 |
| *Orthocladius S* type | Brooks et al. 2007;  Oliver & Roussel 1983 | 13,10,23; 1,1,12; 71,45,5; 0,2,1; 2,2,0 |
| *Paralimnophyes* | Wiederholm 1983 | 10,31,47; 8,27,21; 11,5,4; 0,1,1; 2,2,0 |
| *Paraphaenocladius* | Wiederholm 1983 | 2,2,0; 0,0,0; 0,0,0; 0,0,0; 0,0,0 |
| *Pseudosmittia* | Ferrington & Saether 2011 | 0,0,0; 0,1,0; 1,1,2; 0,1,0; 0,0,0 |
| *Smittia* | Wiederholm 1983 | 0,0,0; 0,1,0; 0,1,5; 0,0,0; 0,0,0 |
| **Other taxa** |  |  |
| Oligochaeta | - | 2,2,54; 1,8,80; 7,9,2; 0,1,3; 0,0,2 |
| Simuliidae | - | 0,2,2; 0,5,2; 0,0,0; 0,0,0; 0,0,0 |
| Collembola | - | 0,0,0; 0,0,0; 3,1,0; 0,0,0; 0,0,1 |
| **Aranea** | - | 0,0,0; 0,0,0; 0,0,1; 0,0,0; 0,0,0 |
| **Ceratopogonidae** |  | 0,1,1; 0,0,0; 0,0,0; 1,0,0; 0,0,0 |
| Clinohelea | Dobson, 2013; Nilsson, A. 1996 | 0,1,0; 0,0,0; 0,0,0; 0,0,0; 0,0,0 |
| Culicoides | Dobson 2013; Nilsson, A. 1996 | 0,0,1; 0,0,0; 0,0,0; 1,0,0; 0,0,0 |
| **Limoniidae** |  |  |
| Chioneinae | Dobson, 2013; Nilsson, A. 1996 | 0,0,0; 1,1,0; 0,1,0; 0,0,0; 0,0,0 |

*Number of specimens per stream in order sites A,B,C for streams Kaerelv, Graenseelv, Unnamed, Aucellaelv, Palnatokeelv, respectively.

References

Brooks SJ, Langdon PG, Heiri O (2007) The Identification and Use of Palaearctic Chironomidae Larvae in Palaeoecology. QRA Technical Guide No. 10, Quaternary Research Association, London. 276pp

Cranston PS (1982) A key to the larvae of the British Orthocladiinae (Chironomidae). Freshwater Biological Association Scientific Publication No. 45. 152pp

Dobson M (2013) Family-level keys to freshwater fly (Diptera) larvae: a brief review and a key to European families avoiding use of mouthpart characters. Freshwater Reviews, 6:1- 32

Ferrington LC, Sæther OA (2011) A revision of the genera Pseudosmittia Edwards, 1932, Allocladius Kieffer, 1913, and Hydrosmittia gen. n. (Diptera: Chironomidae, Orthocladinae), Zootaxa 2849, 1-314

Lindegaard C (2015) Chironomidae (Non-biting midges) In: Böcher J, Kristensen NP, Pape T, Vilhelmsen L In: The Greenland Entomofauna An Identification Manual of Insects, Spiders and Their Allies. Fauna Entomologica Scandinavica, 44, Lund, pp 436–549

Nilsson, A. (1996) Aquatic Insects of North Europe: A taxonomic handbook. Odonata – Diptera, Volume 2. Apollo books, Steenstrup, 440pp

Oliver DR, Roussel ME (1983) The genera of larval midges of Canada. Diptera: Chironomidae. The Insects and Arachnids of Canada 11. Agriculture Canada, Ottawa, 263pp

Sæther OA (1976) Revision of *Hydrobaenus, Trissocladius, Zalutschia, Paratrissocladius* and some related genera. *Bulletin of the Fisheries Research Board of* Canada 195, 1- 287

Wiederholm T (Ed.) (1983) Chironomidae of the Holarctic region. Keys and diagnoses. Part 1 Larvae. *Entomologica Scandinavica* (suppl.) 19, 1-457.
